# Using optical coherence tomography to optimize Mohs micrographic surgery

**DOI:** 10.1038/s41598-024-53457-7

**Published:** 2024-04-17

**Authors:** Sruti S. Akella, Jenna Lee, Julia Roma May, Carolina Puyana, Sasha Kravets, Vassilios Dimitropolous, Maria Tsoukas, Rayyan Manwar, Kamran Avanaki

**Affiliations:** 1 Department of Dermatology, University of Illinois-Chicago, Chicago, IL USA; 2 Present Address: Department of Ophthalmology and Visual Sciences, The Ohio State University Wexner Medical Center, Columbus, OH USA; 3 School of Medicine, University of Illinois-Chicago, Chicago, IL USA; 4 Division of Epidemiology and Biostatistics, School of Public Health, University of Illinois-Chicago, Chicago, IL USA; 5 Department of Biomedical Engineering, University of Illinois-Chicago, Chicago, IL USA

**Keywords:** Basal cell carcinoma, Melanoma

## Abstract

Mohs micrographic surgery (MMS) is considered the gold standard for treating high-risk cutaneous basal cell carcinoma (BCC), but is expensive, time-consuming, and can be unpredictable as to how many stages will be required or how large the final lesion and corresponding surgical defect will be. This study is meant to investigate whether optical coherence tomography (OCT), a highly researched modality in dermatology, can be used preoperatively to map out the borders of BCC, resulting in fewer stages of MMS or a smaller final defect. In this prospective study, 22 patients with BCC undergoing surgical excision were enrolled at a single institution. All patients had previously received a diagnostic biopsy providing confirmation of BCC and had been referred to our center for excision with MMS. Immediately prior to performing MMS, OCT was used to map the borders of the lesion. MMS then proceeded according to standard protocol. OCT images were compared to histopathology for agreement. Histopathologic analysis of 7 of 22 MMS specimens (32%) revealed a total absence of BCC, indicating resolution of BCC after previous diagnostic biopsy. This outcome was correctly predicted by OCT imaging in 6 of 7 cases (86%). Nine tumors (9/22, 41%) had true BCC and required a single MMS stage, which was successfully predicted by pre-operative OCT analysis in 7 of 9 cases (78%). The final six tumors (27%) had true BCC and required two MMS stages for complete excision; preoperative OCT successfully predicted the need for a second stage in five cases (5/6, 83.3%). Overall, OCT diagnosed BCC with 95.5% accuracy (Cohen’s kappa, κ = 0.89 (p-value =  < 0.01) in the center of the lesion. Following a diagnostic biopsy, OCT can be used to verify the existence or absence of residual basal cell carcinoma. When residual tumor is present that requires excision with MMS, OCT can be used to predict tumor borders, optimize surgery and minimize the need for additional surgical stages.

## Introduction

The diagnosis of basal cell carcinoma can be made by a number of non-invasive methods, although biopsy with histopathologic examination remains the gold standard. Handheld dermoscopy, which magnifies an image and uses polarized light, has a reported diagnostic accuracy of 95–99%^1^. However, it has been shown to poorly estimate the overall size of the lesion^2,3^. Reflectance confocal microscopy (RCM) uses a near-infrared low-power laser to image thin sections of skin and has been shown to have high sensitivity (90–100%) and specificity (80–90%). However, handheld RCM imaging has maximal field of view of 0.75 mm^2^ and the depth of image is limited to 200 µm, which can miss aggressive skin cancers and deep margins^4^. Optical coherence tomography (OCT) is a non-invasive imaging technology that uses infrared light to produce real-time, cross-sectional, and *en face* images. Our prior research has demonstrated the use of OCT in examining skin tumors^5–27^. By measuring the backscattering of light from within the skin, it is possible to image the epidermis, dermoepidermal junction, dermis, hair follicles, sweat glands, and blood vessels^28–30^. The handheld OCT probe has a fast acquisition time, a fairly large field of view (e.g., 6 mm $\times$ 6 mm), and achieves an imaging depth up to 1.5 mm^31^. Prior studies have found that OCT is accurate and can improve the sensitivity and specificity of diagnosing BCC over clinical or dermoscopic evaluation alone. Sensitivity and specificity reportedly range from 80 to 90%^32–34^. A meta-analysis of 31 studies calculated a positive predictive value of 79.5% and a negative predictive value of 76.6%^28^.

An advantage of OCT is that it has been studied extensively with regards to diagnosing BCC and therefore a significant body of literature exists that identifies typical malignant features. This can serve to standardize the interpretation of acquired images and reduce inter-observer variability^32,33,35–39^. Recently, a European consensus statement described the top imaging characteristics of the most common types of BCC^40^. For nodular subtype, the top three OCT features were (1) hyporeflective ovoid structures in dermis, (2) hyporeflective peritumoral clefting, and (3) hyporeflective border. The three most important features for superficial BCCs were (1) hyporeflective nests or ovoid structures protruding out of epidermis, (2) hyporeflective bulging into dermis, and (3) epidermal-bound nests. For the group of infiltrative and morpheaphorm BCCs the top three characteristics were: (1) grape-like appearance, (2) multiple nodules separated from epidermis, and (3) smaller and more aggregated nests^40^.

Following diagnosis, the gold standard for treating basal cell carcinoma is surgical excision. In high-risk areas, Mohs micrographic surgery (MMS) in particular has been proven to reduce the recurrence rate of BCC^31^. MMS is the process by which skin surrounding a tumor is removed and immediately analyzed with histopathology; if margins are positive for residual tumor, the surgeon carries out additional excision in the area of positive margins while sparing neighboring tissue with negative margins. Each return to the patient is counted as an additional “stage”. However, this approach poses several challenges. First, it is expensive. Medicare payment data from 2015 to 2017 reports an annual spending of over $500 million dollars for MMS, representing more than 60% of the overall skin cancer surgery expenditure^41^. As additional stages are performed, the cost of MMS directly increases. In 2014, Medicare estimated that the total cost of extra stages alone was $160 million^41^.

Second, the outcomes of Mohs micrographic surgery can be unpredictable. Without a reliable means of preoperative tumor border mapping, there is no way to predict how large the final lesion will be, nor how many stages will be required for its removal. This leaves patients and surgeons alike at the disadvantage of not knowing how large of a surgical defect to expect after the BCC is completely excised.

**Figure 1:**
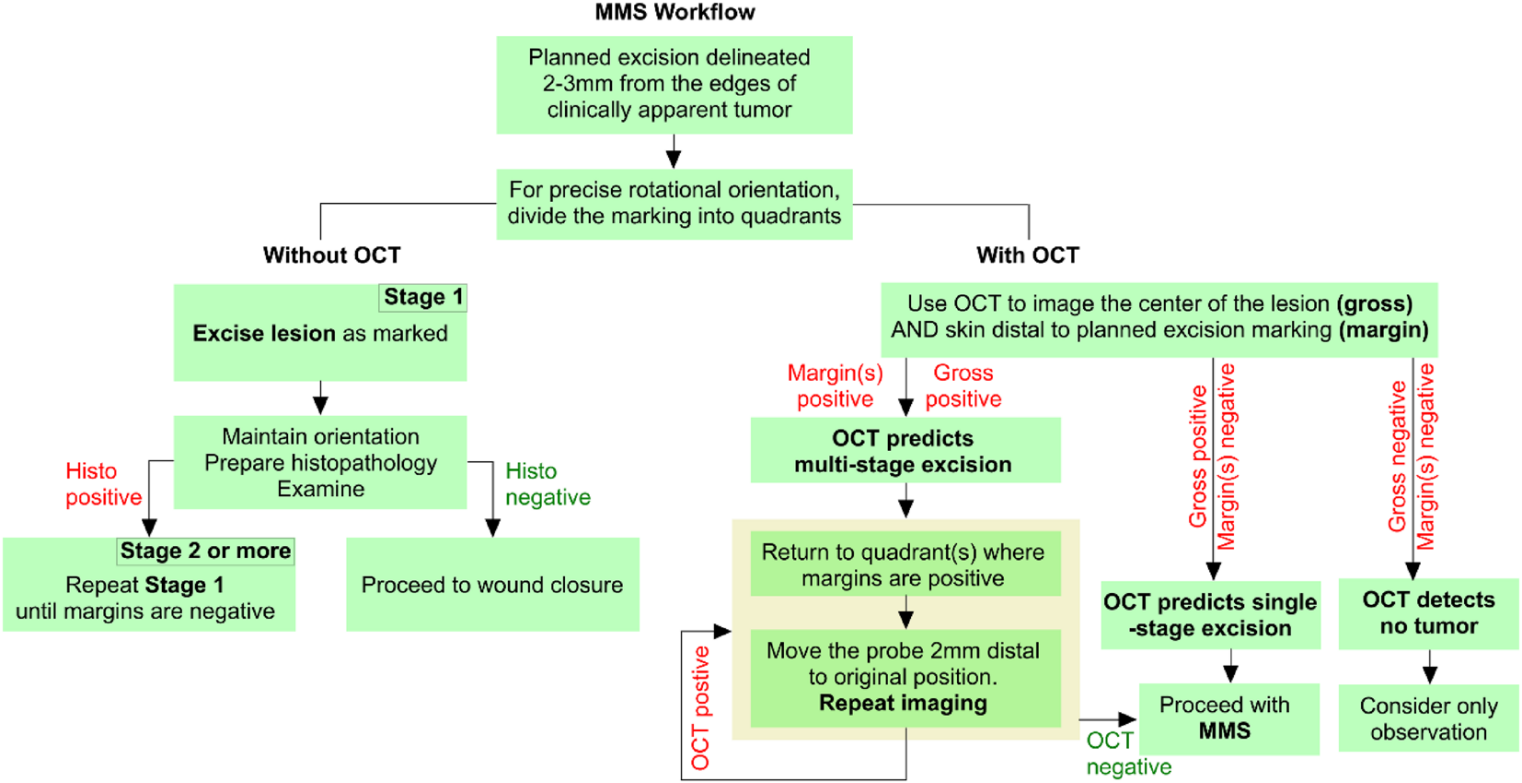
Protocol for Mohs micrographic surgery (MMS) with and without OCT. The left half of the flowchart (“Without OCT”) is the current MMS practice, adapted from Shriner et al.^47^ The right half of the flowchart (“With OCT”) refers to an idealized protocol which incorporates pre-operative OCT tumor mapping to eliminate multiple surgical stages.

To this end, an emerging area of interest is using non-invasive imaging methodologies not just to diagnose BCC but primarily to map out tumor margins before MMS. For the reasons described above, OCT is uniquely suited to margin delineation. By adopting a protocol in which OCT images are captured and analyzed for malignancy before MMS, it may be possible to completely map the tumor boundaries. Surgical excision according to this OCT map could then accomplish complete clearance of the tumor in a single stage, rather than relying on serial stages with repetitive histopathology preparation. A proposed workflow incorporating OCT as compared to existing MMS protocol is shown in Fig. 1. However, only five prior studies have applied this technology to the standard MMS technique, two of which^42,43^ were case reports^42–46^. In these studies, accuracy of OCT in predicting extension of tumor beyond clinical margins ranges from 80 to 100%. There is therefore not enough existing data to determine whether OCT is an accurate and effective method of optimizing Mohs micrographic surgery.

The purpose of this study was: to determine whether there was agreement between OCT-defined margins and histopathology margins, and to determine if mapping out tumor borders preoperatively with OCT can reduce the number of MMS stages.

## Patients and methods

All of the imaging procedures and experimental protocols were approved by the University of Illinois-Chicago’s Institutional Review Board (IRB). All methods were carried out according to the guidelines of the University of Illinois-Chicago’s Institutional Review Board (IRB) and the tenets of the Declaration of Helsinki. Informed consent was obtained from all subjects and/or their legal guardian(s). A total of 25 patients over the age of 18 years with biopsy-proven basal cell carcinoma were recruited for this study. Exclusion criteria were patients with recurrent basal cell carcinoma or BCC arising in a previous surgical site; BCC extending into the orbit; or patients who had undergone previous topical therapies as there was concern that prior manipulation of tissue would result in artifact on OCT imaging.

### Patient preparation

**Figure 2:**
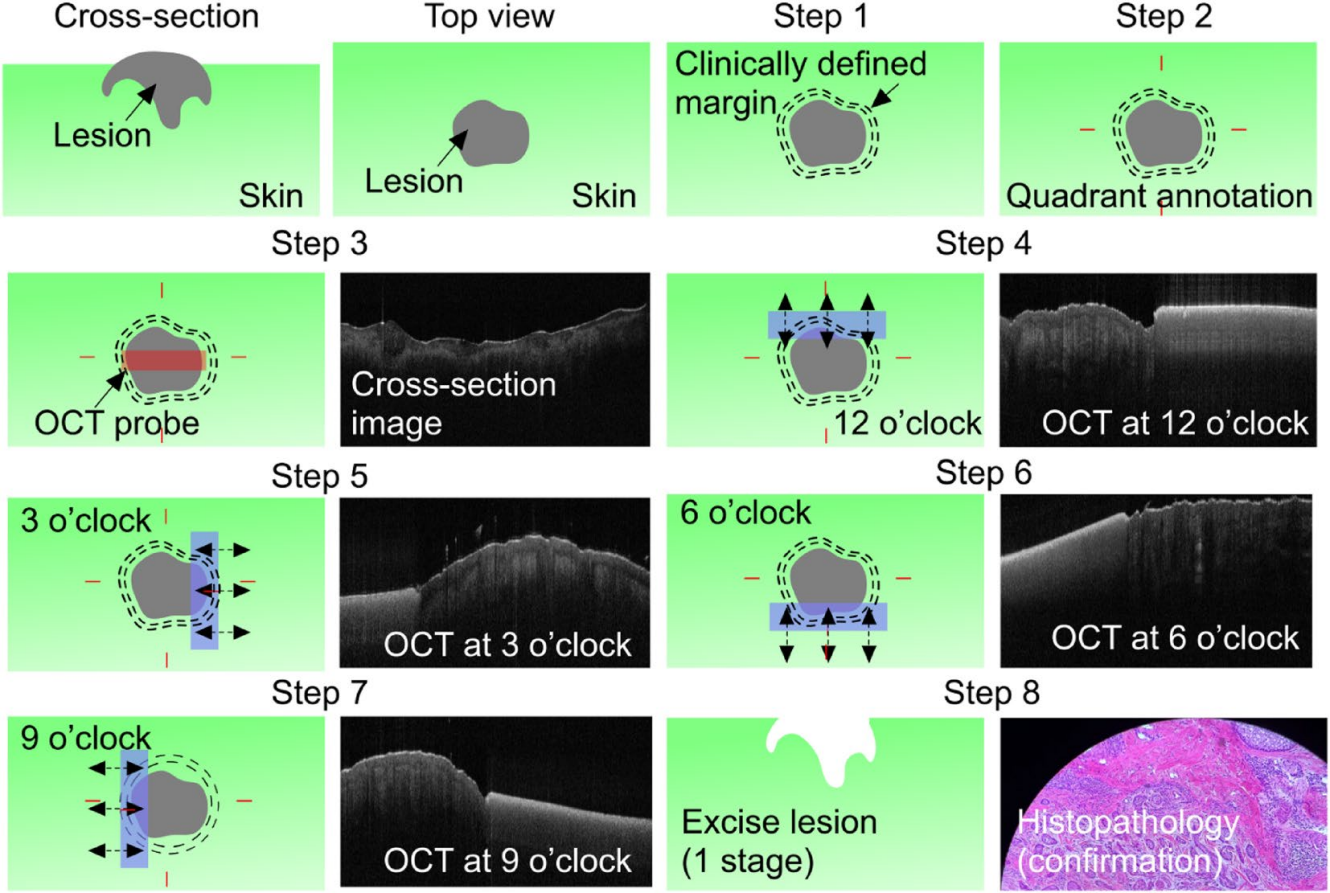
OCT-based imaging process. Step 1: Following gross examination of the lesion, the surgeon marks out the planned primary excision (clinically-defined margin) and divides the lesion into clock hours (Step 2). Step 3: The probe is used to scan the center of the lesion. Steps 4–7: At all defined clock hours, skin distal to the clinically-defined margin is scanned with OCT. Step 8: Following excision of the lesion at the clinically-defined margin, histopathology images are compared to the OCT images acquired in Steps 3–7 (gross image and four clock-margins) for confirmation. Blue rectangle = OCT-opaque marking tape; double-headed arrows = direction of OCT scan perpendicular to the tape*.*

The first macroscopic demarcation of the lesion occurred with the naked eye by the Mohs surgeon, guided by dermoscopy where necessary. A margin of 2–3 mm was then drawn around the lesion by the same Mohs surgeon using a sterile marking pen. The width and length of this first marking were measured in millimeters and recorded as the dimensions of the “clinically-defined margin”. The clinically-defined margin was then divided into distinct sections for OCT imaging by placing a mark at 12 o’clock, 3 o’clock, 6 o’clock, and 9 o’clock (Fig. 2, Steps 1 and 2). For larger lesions, additional clock hours were marked as indicated to provide full coverage.

### Imaging technique

The VivoSight Dx (Michelson Diagnostics, Maidstone, Kent, UK) OCT system uses a handheld 1305 nm scanning infrared laser to image a 6 × 6 mm^2^ area and produces up to 500 cross-sectional slices (frames) per scan with a spatial resolution of 7.5 μm. The system acquired OCT signal using a frame grabber (PCIe-1433, National Instrument), processed signals in real-time using a GPU (GeForce GTX1080, NVIDIA, Santa Clara, California), and used a Dell Precision workstation to coordinate data acquisition, processing, and display.

The first scan with OCT was performed in the center of the lesion (gross) (Fig. 2, Step 3). This was done for diagnostic proof as well as to evaluate the structure of the tumor (i.e. nodules, cysts). The margins were then imaged sequentially starting at 12 o’clock, as follows: an OCT-opaque tape (from the Gentian Violet Surgical Skin Marker with Label, McKesson Corporation, Texas, United States) was placed so that it just covered the clinically defined margin but left the skin distal to the marking exposed (Fig. 2, Step 4, left panel). This created a sharp demarcation border on OCT images such that the photographer was always able to localize, and measure visualized OCT pathology in relation to the clinically-defined margin (Fig. 2, Step 4, right panel). To capture images, the OCT probe was positioned such that the scanning direction of the laser ran perpendicular to the tape (Fig. 2, Step 4). An *en face* scanning image was acquired. This process was repeated at every clock hour that was initially marked (Fig. 2, Steps 4–7).

### Diagnostic criteria

**Figure 3:**
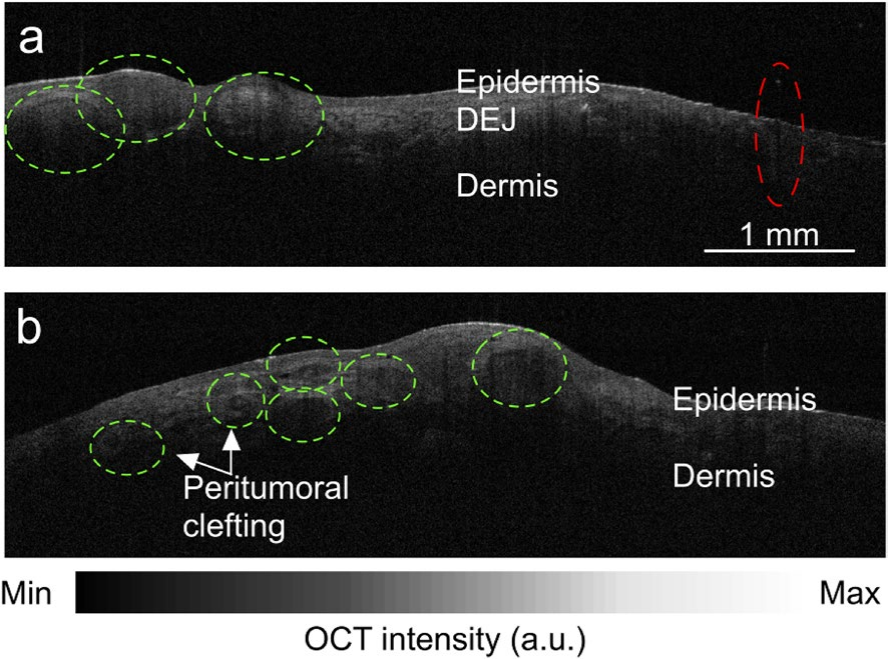
Sample OCT images with malignant features. (**a**) Hyporeflective ovoid structures bulging from epidermis (green dashed line) and shadowing from a typical hair follicle (red dashed line). (**b**) Hyporeflective ovoid structures in the dermis with peritumoral clefting and disruption of the dermoepidermal junction (green dashed line).

After all OCT images were taken, they were despeckled^5^ and a blinded panel of three non-dermatologists evaluated the images for the presence or absence of tumor features using the criteria defined in the 2022 European consensus^40^. The blinded panel consisted of: two medical students and one oculofacial plastic surgery fellow. All three panelists were considered “qualified” after completion of a training session with a member of the bio-imaging PhD laboratory. OCT interpretation was considered “positive” for tumor when any of the following features were present: hyporeflective ovoid structures in dermis, hyporeflective peritumoral clefting, hyporeflective nests or ovoid structures protruding out of epidermis, hyporeflective bulging into dermis, epidermal-bound nests, grape-like appearance of epidermis, multiple nodules separated from epidermis, and smaller and more aggregated nests (Fig. 3). A “positive” OCT finding in any quadrant meant that OCT predicted the need for a “second stage” of Mohs surgery; in other words, it suggested that excising tissue according to the clinically-defined margin would be insufficient to remove all tumor (Fig. 1). A “negative” result meant that none of these features were present, and the imaged clock-hour was therefore considered tumor-free. In these cases, OCT findings were interpreted as meaning that the current planned “first-stage” of excision according to clinically-defined margins would be sufficient to remove all tumor (Fig. 1).

### Standard MMS protocol

After injecting 1% lidocaine with 1:10,000 epinephrine to provide local anesthesia and hemostasis, the surgeon used a #15 Parker-Bard blade to excise the lesion at the clinically defined tumor. The tissue was sent for histologic preparation per MMS guidelines. An additional histology slide was prepared from a sample taken through the center of the excised tissue (“Slice X”) to compare with OCT gross images. Slides were examined by the MMS surgeon and defined as “positive” or “negative” for BCC. Regarding histopathology, a “positive” result meant that the tissue showed characteristic features of BCC such as dark-blue-staining basaloid aggregates with or without peripheral palisading or clefting^48^. A “negative” result meant that these features were absent and the specimen was considered tumor-free. The remainder of the MMS protocol, including closure or additional stages as needed, proceeded according to standard guidelines without further OCT imaging.

### Statistical analysis

Demographic and clinical characteristics were summarized using means (± standard deviation) and percentages for continuous and categorical variables, respectively. Agreement between histopathology and OCT for both initial diagnosis and analysis of quadrants were summarized in two 2 × 2 tables, and Cohen’s Kappa, κ, was reported. A p-value of ≤ 0.05 was considered statistically significant. Data were analyzed using R (R Core Team (2019). R: URL https://www.R-project.org/).

### Ethics approval and accordance statement

All of the imaging procedures and experimental protocols were approved by the University of Illinois-Chicago’s Institutional Review Board (IRB). All methods were carried out according to the guidelines of the University of Illinois-Chicago’s Institutional Review Board (IRB) and the tenets of the Declaration of Helsinki. Informed consent was obtained from all subjects and/or their legal guardian(s).

## Results

Twenty-five patients with biopsy-proven, treatment-naïve BCC undergoing Mohs micrographic surgery were enrolled between October 2021 and March 2022. Three patients were excluded due to processing error (folding) in slide preparation, which precluded accurate histopathological analysis. There were no other exclusions. A total of 22 patients with 22 lesions were included (Table 1).

**Table 1:** Information on patients recruited, characteristics of BCC, and findings by histology and OCT.

Case		Age	Sex	Ethn	Location	Subtype (Primary/Secondary, if applicable)	Longest Horizontal Axis (mm)	Longest Vertical Axis (mm)	Center		12 o’clock		3 o’clock		6 o’clock		9 o’clock	
									Histo	OCT	Histo	OCT	Histo	OCT	Histo	OCT	Histo	OCT
*1*	No BCC	58	M	WNH	Face	Nodular	3	5	−	−	−	−	−	−	−	−	−	−
*2*		73	M	WNH	Face	Nodular/Infiltrating	14	21	−	−	−	−	−	−	−	−	−	−
*3*		76	M	WNH	Face	Nodular	14	21	−	−	−	−	−	−	−	−	−	−
*4*		76	M	HIS	Face	Nodular	15	22	−	−	−	−	−	−	−	−	−	−
*5*		62	M	WNH	Face	Nodular/Infiltrating	14	21	−	** − **	−	−	−	−	−	−	−	** − **
*6*		73	M	HIS	Face	Adenoidal	11	9	−	−	−	−	−	−	−	−	−	−
7*		59	F	WNH	Trunk	Infiltrating	8	8	−	** + **	−	−	−	−	−	** + **	−	−
*8*	True BCC: One Stage	87	M	WNH	Ext	Nodular/Infiltrating	49	61	** + **	** + **	−	−	−	−	−	−	−	−
*9*		47	F	WNH	Face	Nodular/Infiltrating	21	25	** + **	** + **	−	−	−	−	−	−	−	−
*10*		90	M	WNH	Ext	Nodular/Infiltrating	31	34	** + **	** + **	−	−	−	−	−	−	−	−
*11*		87	F	WNH	Face	Nodular/Infiltrating	12	13	** + **	** + **	−	−	−	** − **	−	−	−	−
*12*		65	M	WNH	Face	Nodular/Infiltrating	11	9	** + **	** + **	−	−	−	−	−	−	−	−
*13*		74	M	WNH	Face	Nodular	14	11	** + **	** + **	−	−	−	−	−	−	−	−
*14*		48	F	WNH	Face	Nodular			** + **	** + **	−	−	−	−	−	−	−	−
15*		34	F	WNH	Face	Morpheaform/Infiltrating	7	11	** + **	** + **	−	−	−	** + **	−	−	−	−
16*		65	M	WNH	Ext	Infiltrating	21	55	** + **	** + **	−	−	−	** + **	−	−	−	−
*17*	True BCC: Two Stages	68	M	WNH	Face	Infiltrating	12	10	** + **	** + **	−	−	** + **	** + **	** + **	** + **	−	−
*18*		69	M	WNH	Ext	Infiltrating	11	21	** + **	** + **	** + **	** + **	−	−	−	−	−	−
19**		78	M	WNH	Face	Nodular/Infiltrating	14	11	** + **	** + **	−	−	−	−	−	−	−	−
*20*		58	F	WNH	Face	Nodular/Infiltrating	14	21	** + **	** + **	** + **	** + **	−	−	−	−	−	−
*21*		39	M	WNH	Face	Morpheaform/Infiltrating	20	17	** + **	** + **	** + **	** + **	−	−	−	−	−	−
*22*		64	M	WNH	Face	Nodular	34	41	** + **	** + **	−	−	−	−	** + **	** + **	−	−

*Ethn.* Ethnicity. *WNH* White, non-Hispanic; *HIS* White, Hispanic; *Ext* extremities; + positive for tumor; − negative for tumor; *Histo* histopathology.

*Disagreement between OCT and histopathology at any given location.

**Tumor was detected at a clock hour not imaged by OCT.

The majority of patients were male (73%), Caucasian race (91%), with an average age of 65.9 (± 14.8) years (range 34–90). The majority of lesions were on the face (77%) and were primarily nodular subtype (68%). Demographics are summarized in Table 2.

**Table 2:** Summary characteristics of the patient population and tumor characteristics.

Variable	Summary Statistics (n = 22)
Patient demographics	
Age	65.91 (14.82) [34,90]
Sex, n (%)	
Female	6 (27.27)
Male	16 (72.73)
Ethnicity, n (%)	
White, non-Hispanic	20 (90.91)
White, Hispanic	2 (9.09)
Clinical characteristics	
Location, n (%)	
Extremities	4 (18.18)
Face	17 (77.27)
Trunk	1 (4.55)
BCC primary subtype, n(%)	
Adenoidal	1 (4.55)
Infiltrating	4 (18.18)
Morpheaform	2 (9.09)
Nodular	15 (68.18)
Infiltration	
No	7 (31.82)
Yes	15 (68.18)
Pigmented	
No	20 (90.91)
Yes	2 (9.09)
Clinical features of lesion	
Scaling	
No	14 (63.64)
Yes	8 (36.36)
Presence of sebaceous glands	
No	15 (68.18)
Yes	7 (31.82)
Hair-bearing tissue	
No	16 (72.73)
Yes	6 (27.27)
Ulcerated	
No	14 (63.64)
Yes	8 (36.36)
Dry, flaking skin	
No	20 (90.91)
Yes	2 (9.09)
Bleeding	
No	18 (81.82)
Yes	4 (18.18)
Crusting	
No	18 (81.82)
Yes	4 (18.18)

### Diagnostic accuracy at the center of the lesion

**Figure 4:**
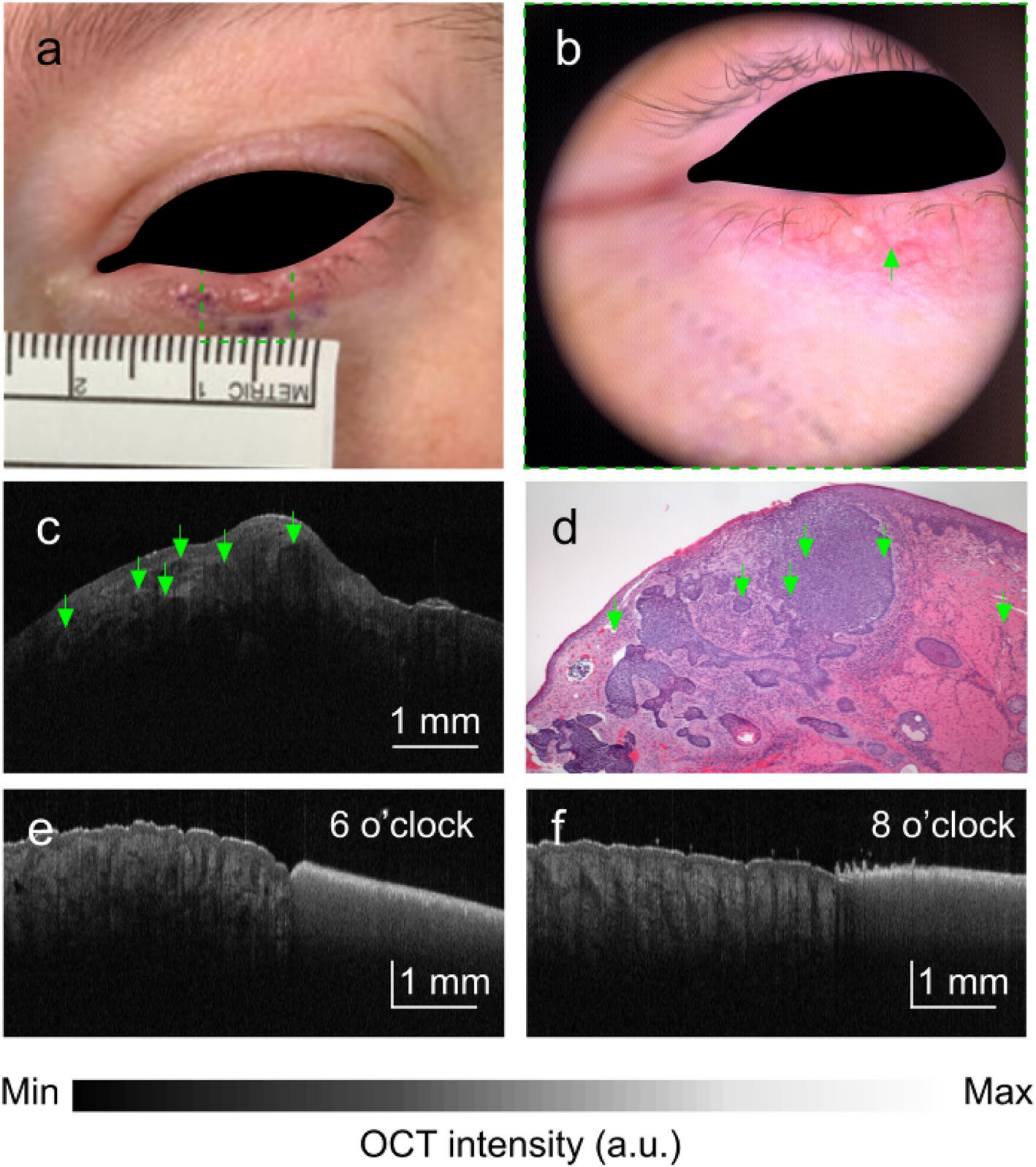
Case #14, nodular BCC of the left lower eyelid cleared in a single stage. (**a**) Gross and (**b**) dermoscopic views. (**c**) Gross OCT imaging showing multiple hyporeflective ovoid structures (green arrows) with corresponding tumor islands seen on histopathology (**d**). OCT images at 6 o’clock (**e**) and 8 o’clock (**f**) margins showing lack of tumor, which was confirmed with histopathology.

Based on histopathologic analysis of Slice X, there were 15 lesions with BCC (Table 1, Cases #8–22) and 7 lesions without BCC (Table 1, Cases #1–7). OCT imaging correctly identified the presence of tumor in all 15 histopathologically positive lesions (sample patient in Fig. 4). In the 7 histopathologically negative lesions, OCT imaging correctly identified the absence of tumor in 6 of 7 cases. In the seventh case of infiltrating BCC (Table 1, Case #7), OCT imaging mistakenly detected the presence of tumor, but histopathology did not show tumor. Overall, agreement between OCT and histopathology was 95.5% (21/22) (Cohen’s kappa, κ = 0.89 (p-value =  < 0.01) (Table 3).

**Table 3:** Agreement between optical coherence tomography and histopathology in the center of the lesion.

		Histopathology	
		Negative	Positive
*OCT*	Negative	6	0
	Positive	1	15
Agreement = 21/22 = 96%			

### Accuracy of OCT in mapping BCC tumor margins

**Figure 5:**
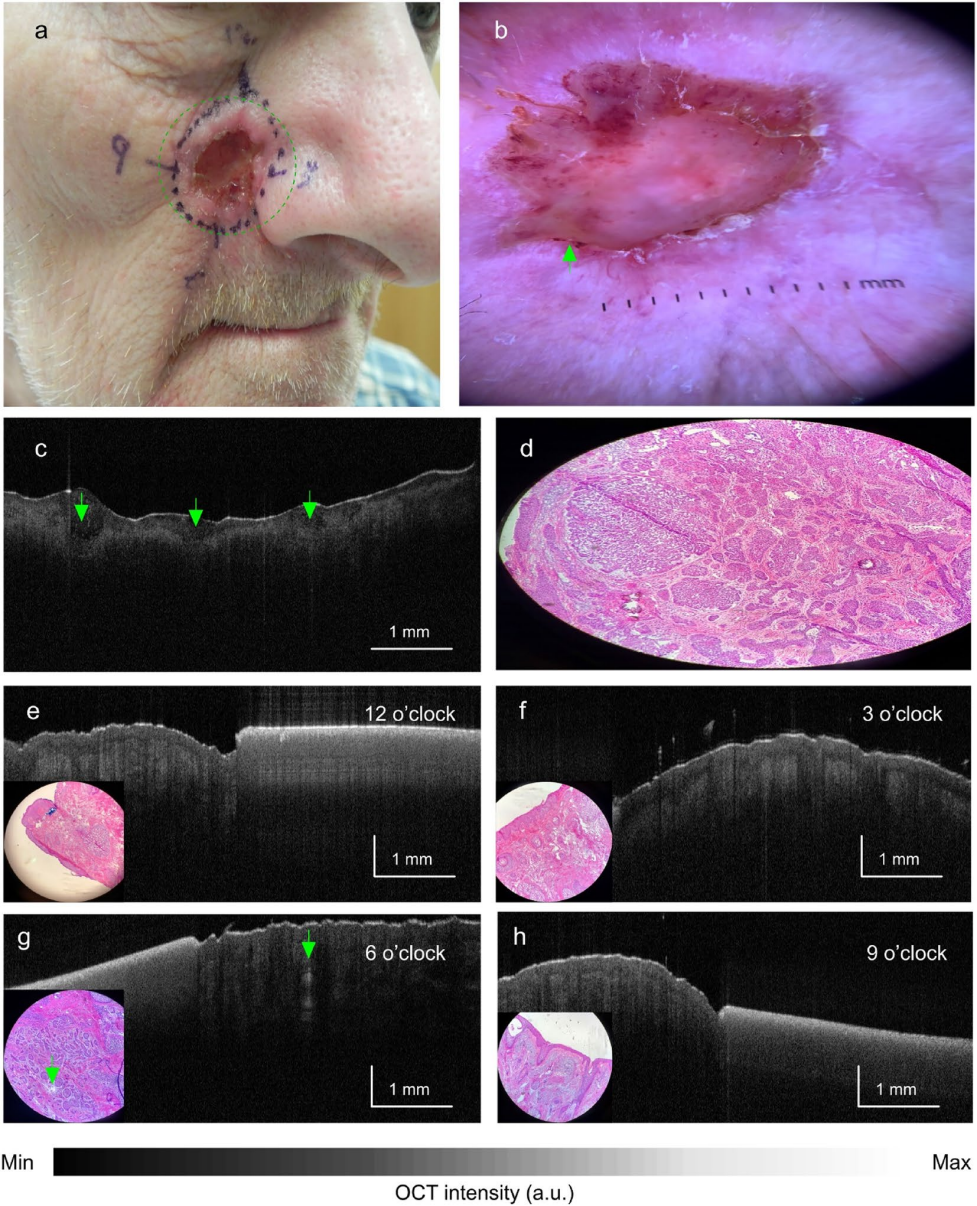
Case #22, nodular BCC of the nasolabial fold in which OCT correctly predicted a second stage. Gross (**a**) and dermoscopic (**b**) views. (**c**) Gross OCT imaging showing multiple hyporeflective ovoid structures in the epidermis (green arrows) with (**d**) corresponding tumor nests on histopathology. OCT images at 12 o’clock (**e**), 3 o’clock (**f**), and 9 o’clock (**h**) showing lack of tumor with corresponding negative histopathology (inset). OCT image at 6 o’clock (**g**) showing hypoechoic tumor nest (green arrow) distal to the clinically-defined margin with corresponding histopathology (inset). A second-stage excision was performed at 6 o’clock.

Of 22 included lesions, six (27%) required an additional (second) MMS stage to achieve total histopathologic clearance (Table 1, Cases #17–22). OCT correctly predicted this outcome in 5 of 6 lesions (sample patient in Fig. 5). For the sixth lesion (Table 1, Case #19, nodular infiltrating subtype), histopathology detected tumor in a clock hour (11 o’clock) that was inside the region imaged by OCT. Because it was not imaged by OCT, no OCT-based prediction was made at this location.

Nine lesions (Table 1, Cases #8–16) were cleared histopathologically in a single MMS stage. OCT correctly predicted this outcome in seven cases (Table 1, Cases# 8–14). All seven of these lesions were primarily nodular subtype. In the other two cases (Table 1, Cases #15–16), OCT mistakenly predicted the need for a second stage which was not required after histopathologic analysis. Case #15 was primarily morpheaform subtype and Case #16 was primarily infiltrating subtype.

**Figure 6:**
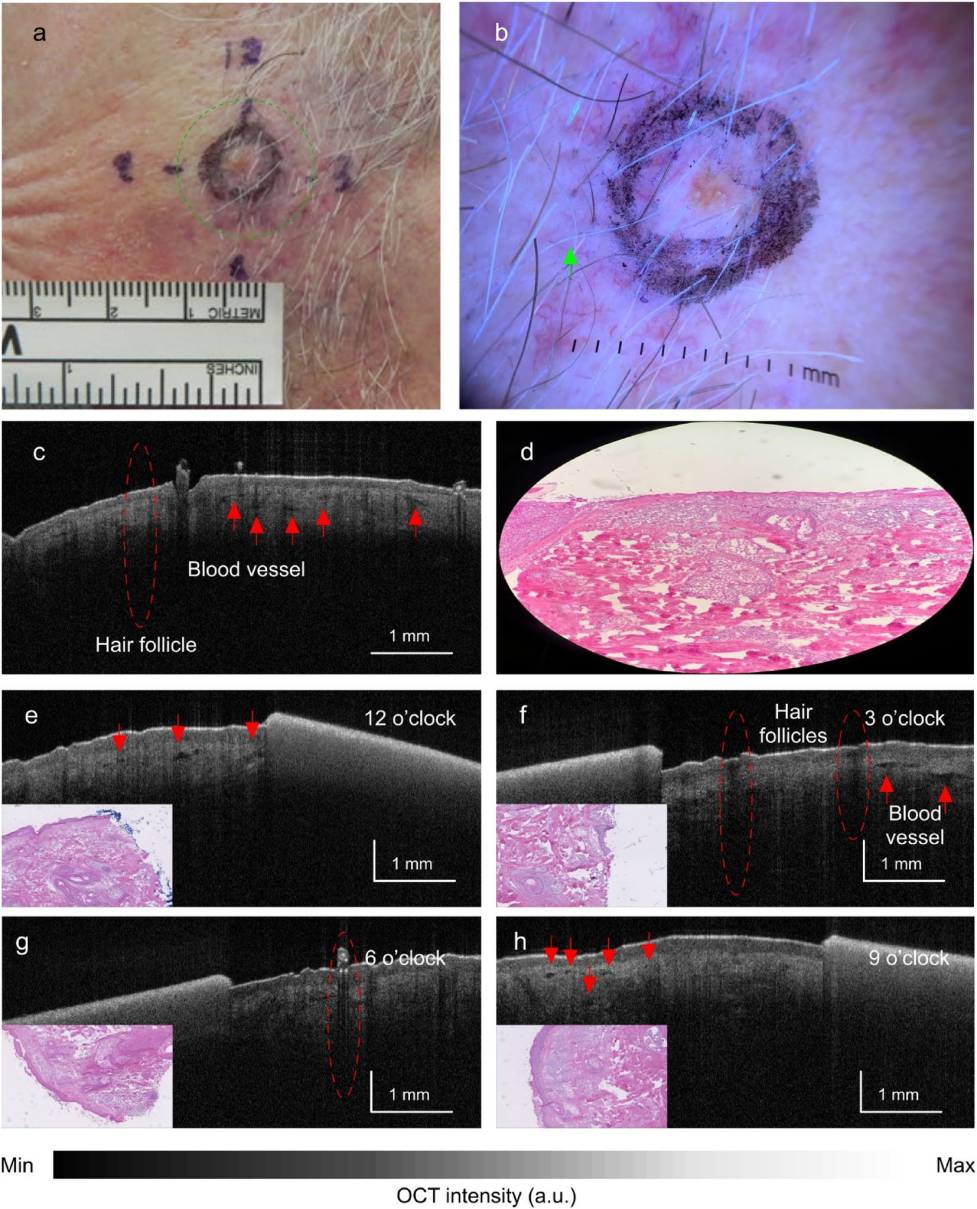
Case #3, prior biopsy site of nodular BCC in left pre-auricular area. Gross (**a**) and (**b**) dermoscopic views. (c) Gross OCT imaging and corresponding histopathology (**d**) showing absence of tumor features. (**e**–**h**) OCT images at four clock hours showing absence of tumor distal to the clinically-defined margin. Histopathology insets confirm the absence of tumor within the clinically-defined margin. Red arrow = blood vessel; red dashed line = hair follicle.

**Figure 7:**
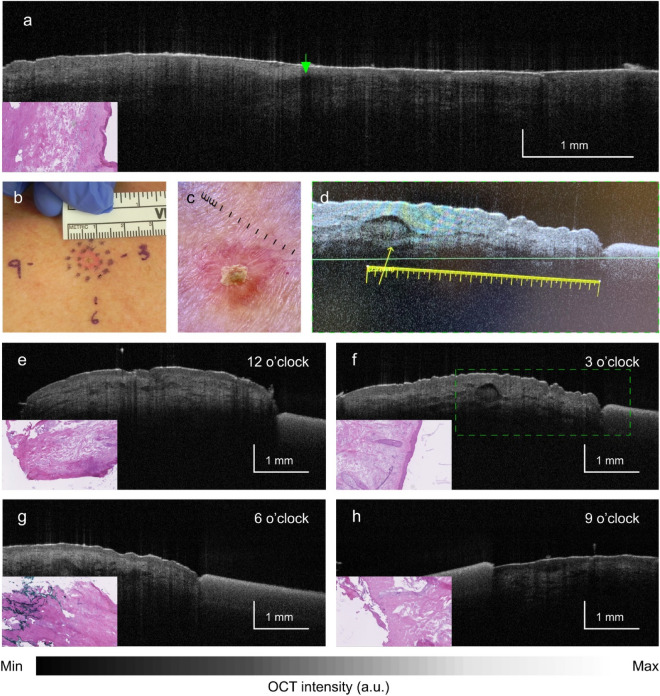
Case #7, previously infiltrating BCC on the trunk found negative by histology and false positive by OCT. (**a**) OCT cross-section of tumor. Green arrow shows hyporeflective ovoid structures in the epidermis. (**b**) Gross and (**c**) dermoscopic views. (**d**) Gross OCT imaging showing hyperreflective ovoid structure (yellow arrow) suggesting tumor (insert from 6 o’clock). (**e**) OCT images at 12 o’clock showing lack of tumor, (**f**) 6 o’clock suggesting tumor, (**g**) 3 o’clock showing lack of tumor and (**h**) 9 o’clock showing lack of tumor.

The remaining 7 lesions (Table 1, Cases #1–7) were confirmed to be negative for BCC based on histopathology both centrally (Slice X) and at all margins of the first-stage MMS specimen (sample patient in Fig. 6). In 6 of the 7 lesions (86%), OCT correctly identified the absence of tumor both centrally and at the margins of the clinically defined border. In the seventh lesion (Table 1, Case #7, infiltrating subtype), OCT mistakenly detected tumor in the center of the lesion and at 6 o’clock. See Fig. 7. However, both Slice X and the first-stage MMS margins were negative histopathologically. Reviewing all the quadrants, agreement between OCT and histopathology was 96.6%, Cohen’s κ = 0.78 (Table 4).

**Table 4:** Agreement between OCT and histopathology at the OCT-defined margins of the lesion.

		*Histopathology*	
		Negative	Positive
OCT	Negative	79	0
	Positive	3	6
Agreement (by quadrant) = 85/88 = 97%			
Agreement (by lesion) = 18/22 = 82%			

The method failed to image/identify one tumor among the 22 cases, leading to under-reporting of tumor in one instance. Three of the OCT quadrant analyses incorrected detected a tumor where none was found by histology.

## Discussion

The advantages of Mohs micrographic surgery are many: in high-risk areas, MMS has been shown to have the highest cure rate (99%) as well as the lowest recurrence rate^31^. The Mohs technique also allows surgeons to examine histopathologic margins intraoperatively and to conserve as much normal tissue as possible. However, MMS is also expensive: in 2017, the total Mohs expenditure paid by Medicare Part B alone was over $537 million, which represented a steady increase from total expenditure in 2014^41^. As the incidence of BCC continues to rise, this number is likewise projected to increase. It is therefore of great interest to decrease the overall cost of MMS and wait-times for MMS, and one projected method of doing so is by reducing the number of procedural stages. It has been estimated that a mere 10% reduction in the average number of stages per case could save $36 million per year^2,49^.

To this end, our study shows promising results for the use of OCT to accurately define tumor perimeter, therefore minimizing the number of MMS stages. Of nine single-stage MMS lesions, OCT correctly predicted histopathology results in 7 (78%) cases. Of 6 two-stage MMS lesions, OCT correctly predicted histopathology results in 5 (83%) cases. In addition, OCT correctly predicted the complete absence of tumor in 6 of 7 cases (86%). In other words, use of OCT to draw tumor margins would have led to 5 patients going from 2 MMS stages down to 1 MMS stage and would have recommended no surgery (but repeat biopsy) for 6 additional patients (from 1 MMS stage to 0 MMS stage). OCT would have reduced the number of stages (and cost) for 50% of the 22 patients enrolled in this very preliminary study. To be fair, OCT would also have unnecessarily increased surgical margins in 2 of the 22 patients (10%), and if OCT recommendations had been followed in their cases, they would still have only required 1 MMS stage but would have experienced marginally larger excisions (based on 2 mm margins increased in the relevant quadrant, for these tumors, excisional size would have increased by approximately 28-35mm^2^). Of note, this study intentionally used personnel with less OCT experience in order to assess the accuracy of this technique in an “early adoption” scenario where there may not be an expert reader available. This certainly could have affected our accuracy.

The absence of tumor in 36.8% of MMS specimens (Table 1, Cases # 1–7) appears to suggest that BCC regressed in the time between prior diagnostic biopsy and presentation for Mohs surgery. It has previously been postulated that the combination of biopsy with the body’s natural inflammatory response can eradicate BCC, and indeed prior histopathologic studies have confirmed that as many as 22–25% of MMS specimens are negative for tumor^50–52^. Absent OCT findings may therefore be an indicator to rebiopsy or observe a small biopsied lesion rather than perform MMS, particularly if the location is in a cosmetically risky area. This may be the most groundbreaking application of OCT.

However, it must be noted that our rate of BCC regression after biopsy (36.8%) was higher than the quoted rate in the literature (22–25%)^26,27^. Reviewing our dataset, the reason for this may be two-fold: first, the majority of our lesions measured less than 300mm^2^ in total area biopsied (e.g. smaller than ~ 2 cm diameter if round), and it could be the case that smaller BCCs have a higher chance of spontaneous regression following biopsy. Second, it is possible that a single cut through the center (“slice X”) is insufficient to make a determination about BCC regression. In future studies, we plan to alter the protocol by sampling one central cut for every 3 mm in order to eliminate this concern. However, the sensitivity and specificity of diagnosing BCC from OCT has been well-established by numerous prior studies to be in the range of at least 80–90%^32–34^. Therefore, we believe that, with further studies, there can be confidence in proclaiming an area tumor-free if there are absent findings on OCT. For example, we did not have any instances of a “negative” slice X OCT interpretation that were associated with either a “positive” slice X histopathology finding or with a second stage, suggesting that even our single slice X carries some degree of accuracy.

**Figure 8:**
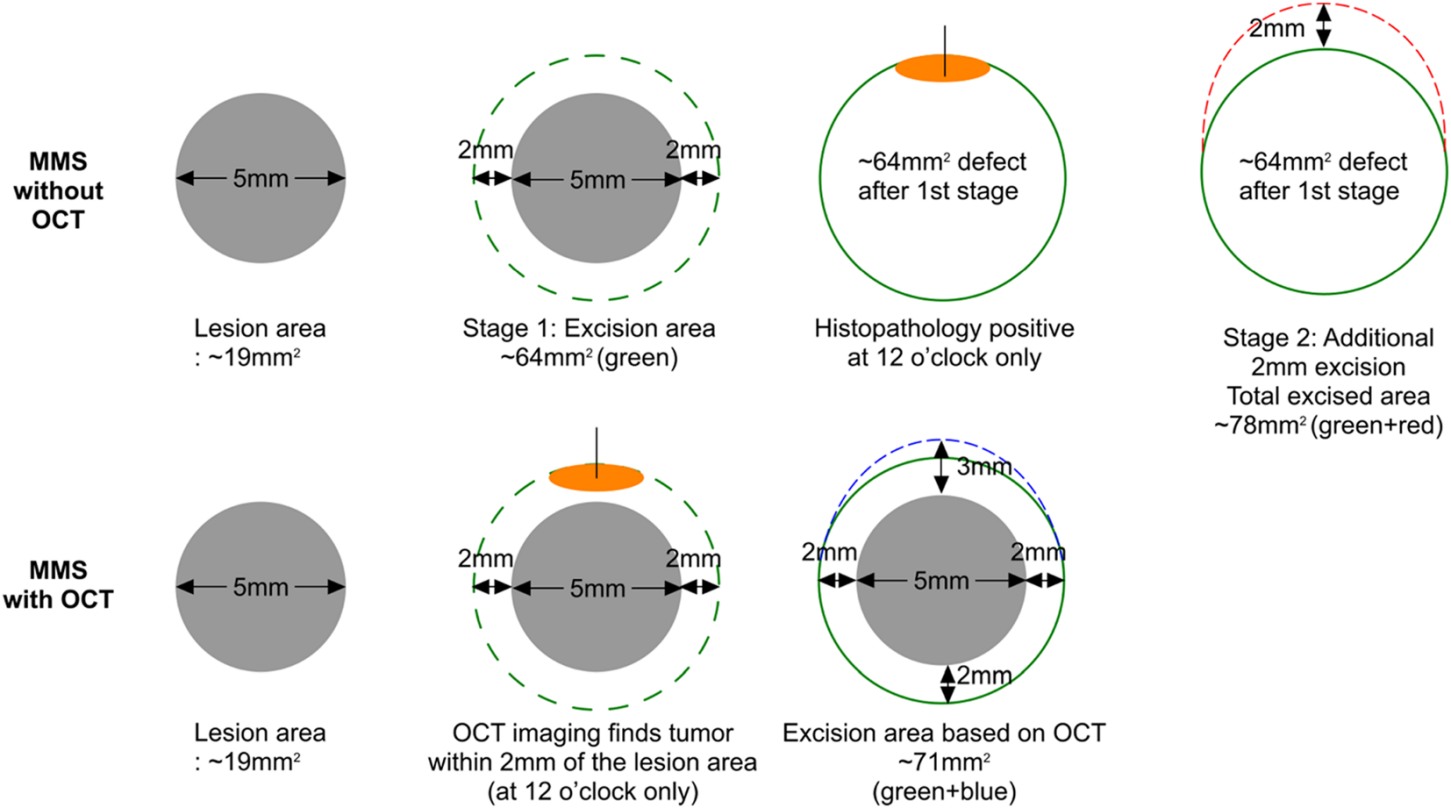
Schematic showing comparison between the amount of tissue excised without OCT (top) and with OCT (bottom) for a hypothetical lesion of 5 mm diameter.

The application of OCT to the existing MMS protocol may provide a benefit of reducing the number of stages, and in cases of OCT-negative lesions, may eliminate the need for Mohs surgery altogether. However, an additional effect of the OCT protocol is that it may serve to actually reduce the amount of tissue that is excised, which is an enormous advantage when considering the patient’s functional and cosmetic outcomes after reconstruction. Figure 8 illustrates how the elimination of multiple stages with OCT (and therefore, multiple 2-3 mm safety margins), can result in a smaller final defect.

OCT has limitations as well, which are illustrated in this study. In four of 22 lesions (18%), OCT had worse specificity than histology. In the single-stage MMS group (Table 1, Cases #8–16), two lesions yielded false positives, meaning that OCT mistakenly detected tumor at margins where there was none based on histopathology. In these instances, excision based on OCT-defined margins would have had the unwanted effect of removing more tissue than necessary. In the two-stage MMS group (Table 1, Cases #17–22), OCT failed to predict a second stage in one case because imaging was not performed at the eleven o’clock hour, where a histopathologic tumor was found. In this instance, excision and histopathologic analysis according to the OCT-defined margin would still have necessitated a second-stage excision. This finding suggests that our methodology of imaging only at certain clock hours (in other words, imaging outside a rectangular box), while expedient, is incomplete. This could be overcome by using a combined OCT/dermoscopy setup to overlay the exact location of the surgeon's markings around the lesion for each OCT image collected. In this case, OCT could image the entire area outside the proposed excision margin and not simply the four quadrants outside the lesion, which was the method performed here. Finally, in the group without tumor (Table 1, Cases #1–7), there was one instance of OCT providing a false positive. In this case, excision based on OCT mapping would have resulted in a larger defect than necessary. This is consistent with a recent Cochrane review which estimated that OCT, when applied to a hypothetical population of 1000 BCC lesions, would miss 31 BCCs (91 fewer than would be missed by visual inspection alone and 53 fewer than would be missed by visual inspection plus dermoscopy), and OCT would lead to 93 false‐positive results for BCC (a reduction in unnecessary excisions of 159 compared to using visual inspection alone and of 87 compared to visual inspection plus dermoscopy)^53^.

In our four reported cases of disagreement with OCT and histopathology, the location of the lesion (face, trunk, or extremities) did not appear to affect diagnostic accuracy. However, all four cases of OCT misdiagnosis were infiltrating or morpheaform subtypes; the remaining 18 cases in which OCT was accurate were primarily nodular subtype. Thus, the BCC subtype may be taken into account when interpreting OCT images. Based on our dataset, the diagnosis of nodular subtype by OCT is most accurate. Other subtypes proved more challenging to diagnose with OCT imaging for two reasons: first and primarily, these more aggressive BCCs tend to produce scaling, crusting, ulceration, and other skin changes which create artifact and shadowing on OCT which can mask the tumor islands; second, whereas nodular subtypes typically produce hyporeflective ovoid structures which are relatively easy to identify, the non-nodular subtypes often display less evident OCT imaging findings, such as grape-like clusters or poorly-circumscribed hyporeflective areas. This finding was also seen in the 2020 study by Sinx KAE et al*.*^33^ which found a higher specificity in diagnosing nodular BCC over other types. Therefore, we suggest that technique is probably most reliable for use in nodular subtypes. With the addition of further prospective studies, we can then assess the relative risk and number needed to treat for other subtypes.

A final consideration of OCT imaging is cost, both in terms of finances (the average OCT machine is valued at approximately $85,000) and manpower (OCT imaging and analysis of a single lesion can take up to 30 min). With regards to cost, some companies such as Lumedica Inc. have recently manufactured low-cost OCT systems (less than $10,000), but they are not yet approved for dermal use. In addition, we may also model reimbursements for an OCT-MMS protocol off the current reflectance confocal microscopy (RCM) imaging protocol, mainly because RCM has a current procedural terminology (CPT) code (96,932, valued at 3.82 RVUs), which would offset the financial burden of this additional work as well as the cost of purchasing this machine^54^. In terms of manpower, we believe that this protocol has real potential to reduce the number of stages that are performed. The histopathological preparation and analysis of each stage takes approximately 30–45 min, and in some institutions requires the hiring and payment of a traveling Mohs technician team. Therefore, the increased time required to capture and analyze OCT images would be offset by the reduction in MMS stages. Finally, Medicare estimates that the cost of additional MMS stages in the United States is upwards of $160 million^41^.

OCT shows early promise as a non-invasive method to delineate tumor margins prior to MMS. RCM has already been approved as a diagnostic tool in the field of dermatology and it would be valuable to perform a study analyzing lesions scheduled for MMS by both OCT and RCM to better understand the strengths and weaknesses of implementing these modalities and/or to improve overall accuracy.

It should be noted that this research has a long evaluation period before it could be adopted or established to help MMS without confirmation from histopathology results. We anticipate that histopathologists will be the key interpreters of OCT data—thus, histopathologists could in the future look at tissue slice-equivalents (OCT images) rather than looking at tissue slices directly. To fully establish the application of OCT in MMS, the resolution of OCT should also be greatly improved. In ongoing research, including in our group, the capability of OCT to produce histology-grade images for use in pathology labs is being explored. In the future, we hope that this technology will be able to reduce the discordance rate among pathologists.

Incorporating image enhancement techniques^5,9, 10, 55–68^ into OCT can improve the quality of the images, thereby optimizing the utilization of their morphological information for more effective margin detection in applications.

## Data Availability

The datasets used and/or analyzed relevant to the presented study are available from the corresponding author on reasonable request.
